# Loxapine inhibits replication of hepatitis A virus *in vitro* and *in vivo* by targeting viral protein 2C

**DOI:** 10.1371/journal.ppat.1012091

**Published:** 2024-03-13

**Authors:** Mami Matsuda, Asuka Hirai-Yuki, Osamu Kotani, Michiyo Kataoka, Xin Zheng, Daisuke Yamane, Masaru Yokoyama, Koji Ishii, Masamichi Muramatsu, Ryosuke Suzuki

**Affiliations:** 1 Department of Virology II, National Institute of Infectious Diseases, Tokyo, Japan; 2 Management Department of Biosafety, Laboratory Animal, and Pathogen Bank, National Institute of Infectious Diseases, Tokyo, Japan; 3 Pathogen Genomics Center, National Institute for Infectious Diseases, Tokyo, Japan; 4 Department of Pathology, National Institute of Infectious Diseases, Tokyo, Japan; 5 Department of Microbiology and Cell Biology, Tokyo Metropolitan Institute of Medical Science, Tokyo, Japan; 6 Department of Quality Assurance, Radiation Safety, and Information System, National Institute of Infectious Diseases, Tokyo, Japan; 7 Department of Infectious Disease Research, Foundation for Biomedical Research and Innovation at Kobe, Kobe, Japan; 8 Department of Biological Science and Technology, Faculty of Advanced Engineering, Tokyo University of Science, Tokyo, Japan; Rutgers University: Rutgers The State University of New Jersey, UNITED STATES

## Abstract

No antiviral drugs currently are available for treatment of infection by hepatitis A virus (HAV), a causative agent of acute hepatitis, a potentially life-threatening disease. Chemical screening of a small-compound library using nanoluciferase-expressing HAV identified loxapine succinate, a selective dopamine receptor D2 antagonist, as a potent inhibitor of HAV propagation *in vitro*. Loxapine succinate did not inhibit viral entry nor internal ribosome entry site (IRES)-dependent translation, but exhibited strong inhibition of viral RNA replication. Blind passage of HAV in the presence of loxapine succinate resulted in the accumulation of viruses containing mutations in the 2C-encoding region, which contributed to resistance to loxapine succinate. Analysis of molecular dynamics simulations of the interaction between 2C and loxapine suggested that loxapine binds to the N-terminal region of 2C, and that resistant mutations impede these interactions. We further demonstrated that administration of loxapine succinate to HAV-infected *Ifnar1*^*-/-*^ mice (which lack the type I interferon receptor) results in decreases in the levels of fecal HAV RNA and of intrahepatic HAV RNA at an early stage of infection. These findings suggest that HAV protein 2C is a potential target for antivirals, and provide novel insights into the development of drugs for the treatment of hepatitis A.

## Author summary

Hepatitis A outbreaks have occurred world-wide, not only in developing countries but also in developed countries, resulting in 15,000–30,000 mortalities annually. However, no antiviral drugs currently are available for the treatment of hepatitis A virus (HAV) infection. Using a reporter-based recombinant HAV, a chemical library of pharmacologically active compounds was screened to identify drugs that specifically inhibit HAV propagation. We show here that loxapine succinate, a tricyclic antipsychotic medication that has been approved by the US FDA for the treatment of schizophrenia, is a potent inhibitor of HAV replication. The effect of loxapine on HAV appears to be independent of the dopamine D2 receptor (the target of the antipsychotic activity), instead corresponding to affinity for the HAV 2C protein, as demonstrated by molecular analysis of loxapine-resistant virus as well as *in silico* modeling. We further showed that administration of loxapine succinate to HAV-infected *Ifnar1*^*-/-*^ mice (which lack the type 1 interferon receptor) resulted in decreases in the levels of fecal HAV RNA and of intrahepatic HAV RNA at an early stage. Together, these results suggest a new target for the development of therapeutics for the treatment of HAV-infected patients.

## Introduction

Hepatitis A virus (HAV) is an important causative agent of acute liver disease in humans; the virus is transmitted via the fecal-oral route through ingestion of contaminated food and water, or through person-to-person contact [1]. The World Health Organization (WHO) estimates that more than 100 million people world-wide are infected with HAV, resulting in more than 15,000–30,000 mortalities from hepatitis A annually. Recent hepatitis A outbreaks have occurred not only in developing countries but also in developed countries around the globe [2–5]. Although a vaccine consisting of inactivated virus is highly efficacious in preventing HAV infection, no HAV-specific antiviral drug is available for the treatment of those already infected.

HAV, a member of the genus *Hepatovirus* within the family *Picornaviridae*, is a small, quasi-enveloped, positive-sense, single-stranded RNA virus. The HAV genome encodes a single polyprotein that is cleaved, post-translationally, into the structural proteins VP4, VP2, VP3, and VP1-pX, all of which contribute to the assembly of viral particles; and the nonstructural proteins 2B, 2C, 3A, 3B, 3C, and 3D, which are involved in viral replication. The nonstructural 2C protein is the most conserved polypeptide among the *Picornaviridae*, and serves as a multifunctional protein that is essential for viral replication. HAV 2C associates with intracellular membranes via its N-terminal domain; the protein has ATPase and RNase activities, but (unlike the 2C homolog from enterovirus 71 (EV71)) lacks helicase activity [6]. The RNase activity of HAV 2C is specific for single-stranded RNA (ssRNA), independent of ATPase activity, and is thought to be critical for RNA replication [6]. The nonstructural HAV 3C protein is a cysteine proteinase that is responsible for most cleavage events within the viral polyprotein. This protease activity also contributes to subverting the host innate immune responses. The nonstructural HAV 3D protein is a viral RNA-dependent RNA polymerase and constitutes the catalytic core of the viral replicase complex.

To identify new inhibitors of HAV replication, we screened a 1280-compound library (LOPAC) using a NanoLuc Luciferase (NLuc)-expressing HAV (HAV-NLuc) [7]. This screen identified loxapine succinate as an inhibitor of HAV replication. Loxapine succinate is a dopamine receptor D2 (DRD2) antagonist, and has been approved by the United States Food and Drug Administration (US FDA) for use as a therapeutic agent in patients with schizophrenia. Loxapine also has been reported to possess potent antibacterial activity against intracellular microbes, including intracellular *Salmonella typhimurium*, methicillin-resistant *Staphylococcus aureus*, *Yersinia enterocolitica*, and macrophage-located *Shigella flexneri*; loxapine’s antibacterial effects are mediated by inhibition of bacterial efflux pumps, which are required for the survival of these microbes in host cells [8].

Blind passage of HAV in the presence of loxapine succinate yielded viruses containing mutations in the 2C-encoding region. These mutations abrogated viral susceptibility to loxapine succinate. Furthermore, molecular dynamics simulations suggested that the compound binds to the N-terminal domain of 2C, and that the mutant proteins exhibit decreased affinity for the compound. Together, these results indicated that the antiviral activity of the compound may be independent of loxapine’s affinity for DRD2. Additionally, we showed that administration of loxapine succinate in HAV-infected *Ifnar1*^*-/-*^ mice, a model of HAV infection [9,10], resulted in decreased fecal levels of HAV RNA and of intrahepatic HAV RNA.

## Results

### Identification of a novel small-molecule inhibitor of HAV replication

**Fig 1:**
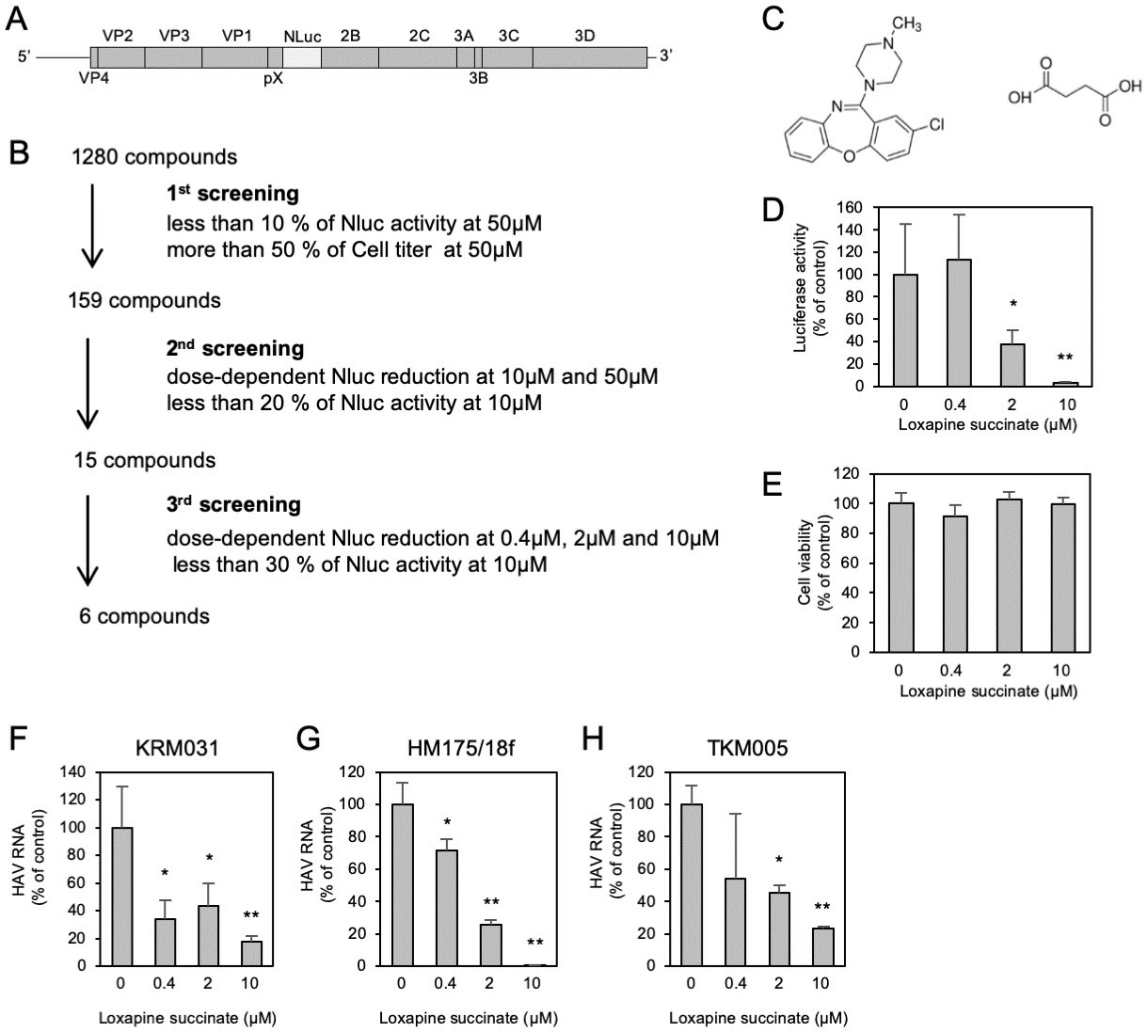
Loxapine succinate inhibits HAV propagation in Huh7.5.1 cells. (A) Schematic of the HAV/Nluc genome with *Nluc* sequence inserted at the pX-2B junction site of the HM175/18f strain sequence. (B) Summary of results from screening of the Library of Pharmacologically Active Compounds (LOPAC1280) for inhibitors of HAV infection. (C) Structural formula of loxapine succinate. (D, E) Cells were infected with HAV/Nluc in the presence and absence of loxapine succinate at the indicated concentrations. Nluc activity (D) and cell viability (E) were determined at 72 h post-infection (hpi). (F-H) Huh7.5.1 cells were infected with 2.0 × 10^2^ genome equivalents (GE) /cell of the HAV KRM031 (genotype IA) (F), HM175/18f (genotype IB) (G), or TKM005 (genotype IB) (H) strains in the presence of loxapine succinate (0.4, 2, or 10 μM); at 72 hpi, the cultures were washed to remove extracellular virus, and culturing was continued in the presence of compound. At 100 hpi, extracellular HAV RNA was quantified by RT-qPCR. Statistical significance was evaluated using a two-tailed non-paired Student’s t-test. * P < 0.05, ** P < 0.01 (vs. control).

In an effort to identify small-molecule inhibitors of HAV infection, we employed a chemical screening assay using a NanoLuc luciferase (Nluc)-expressing HAV (HAV/NLuc) [7], as shown in Fig 1A. Huh7.5.1 cells were seeded into 96-well plates and infected with HAV/NLuc in the presence of compounds. Nluc activity was analyzed at 3 days post-infection (dpi). This assay allowed the detection of inhibitors of viral attachment/entry, translation, replication, and assembly/egress. In parallel, potential cytotoxic effects of the drugs were assessed by quantifying ATP levels (using the CellTiter-Glo Kit; Promega, Madison, WI, USA) in exposed cells.

In the initial screen, primary hits were defined as 159 compounds that (at concentrations of 50 μM) decreased luciferase activity more than 10-fold while exhibiting little or no cytotoxicity (less than 50%). Such primary hits then were evaluated further for reproducibility, dose dependency, and cytotoxicity, and 6 compounds were identified after 3 rounds of screening (Fig 1B). Among these 6, we focused on loxapine succinate as the highest-potency compound that provided decreased Nluc activity without apparent cytotoxicity (Fig 1C–1E). Loxapine succinate is the succinate salt form of loxapine, a DRD2 antagonist used clinically for the treatment of schizophrenia. To confirm the anti-HAV effect of loxapine succinate, we tested the compound against three HAV strains (KRM031: genotype IA, HM175/18f: genotype IB, and TKM005: genotype IB). As shown in Fig 1F–1H, treatment with loxapine succinate consistently prevented the accumulation of viral RNA in the spent medium of cells infected with each of these strains of HAV, consistent with the original results obtained with HAV/NLuc.

### Antiviral activity of loxapine succinate against other RNA viruses

To determine whether the antiviral effect of loxapine succinate is specific for HAV, we tested the compound’s effects on enterovirus D68 (EV-D68), a distantly related picornavirus; as well as on dengue virus (DV) and hepatitis C virus (HCV), hepatotropic members of the *Flaviviridae* family. As shown in S1 Fig, exposure to loxapine succinate had no effect on the growth of EV-D68 or DV type 1, suggesting that loxapine succinate is not a general antiviral for members of the family *Picornaviridae* or *Flaviviridae*. Interestingly, HCV propagation was decreased by exposure to loxapine succinate, suggesting that HAV is not the only virus inhibited *in vitro* by treatment with loxapine succinate.

### Loxapine succinate specifically inhibits the HAV genome replication step

**Fig 2:**
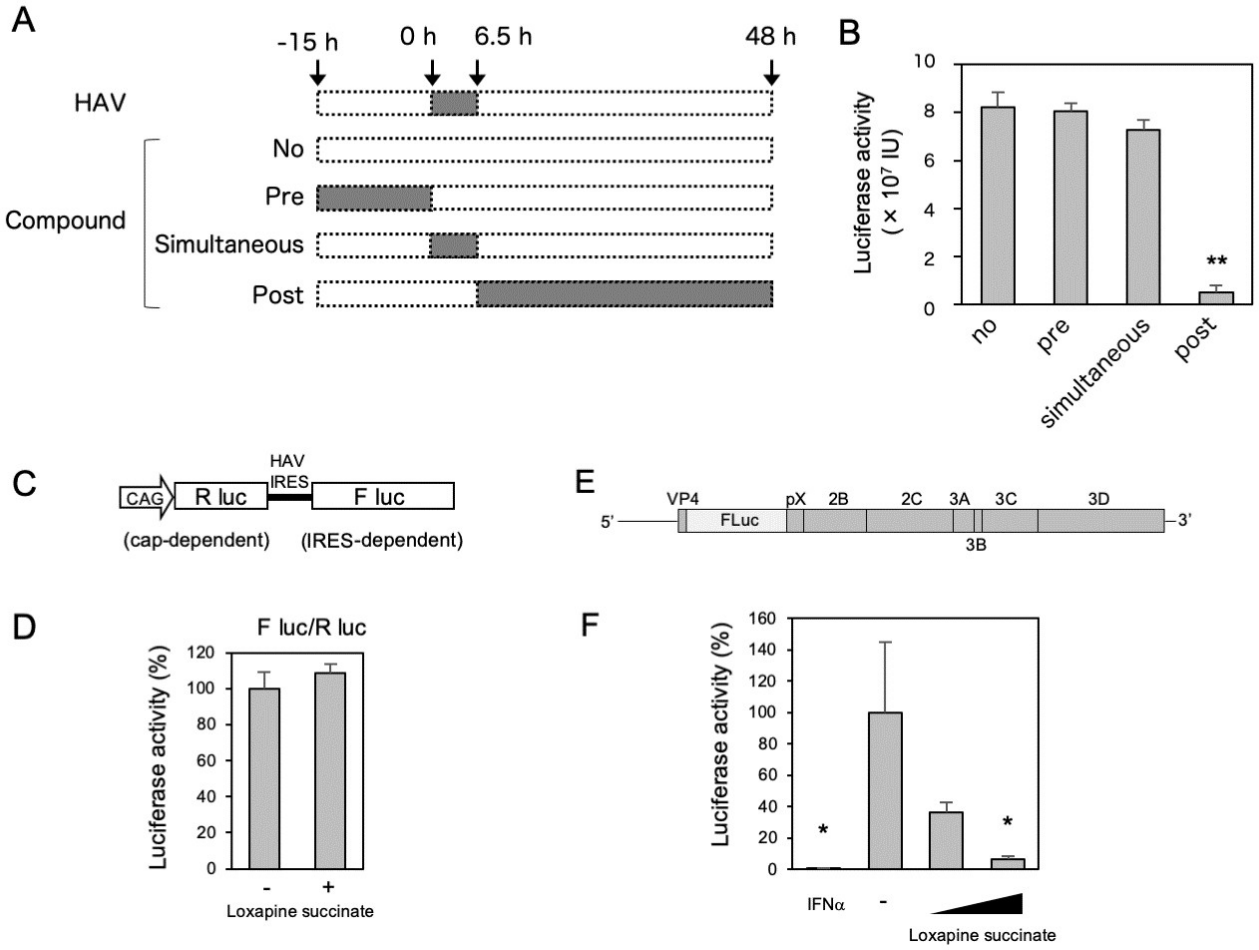
Loxapine succinate inhibits HAV infection via an RNA replication step. (A) Schematic representation of the schedule for loxapine exposure in Huh7.5.1 cell-based HAV infection. No: no treatment. Pre: Huh7.5.1 cells were pretreated with loxapine succinate (10 μM) for 15 h and then inoculated with HAV in the absence of compound. After washing to remove extracellular HAV (and compound), cells were cultured with medium in the absence of compound for up to 48 h, and HAV infection was quantified by measuring Nluc activity in cell lysates. Simultaneously: Huh7.5.1 cells were inoculated with HAV in the presence of the compound (10 μM); at 6.5 hours post-infection (hpi), cells were washed to remove extracellular HAV (and compound) and then cultured with medium in the absence of compound for up to 48 h. HAV infection was quantified by measuring Nluc activity in cell lysates. Post: Huh7.5.1 cells were inoculated with HAV; at 6.5 hpi, cells were washed to remove extracellular HAV and then cultured with medium containing compound (10 μM) for up to 48 h. (B) At 48 hpi, the cells were harvested and NLuc activity was determined. The data are presented as the mean ± SD from three independent experiments. Statistical significance was determined using a two-tailed non-paired Student’s t test. *, P < 0.05 (compared to no treatment). (C) Schematic diagram of the bicistronic expression plasmid used in this study. (D) Huh7.5.1 cells were transfected with the bicistronic reporter plasmid. Supernatant was replaced 6 h posttransfection with medium containing (10 μM) or lacking loxapine succinate. The firefly luciferase (FLuc) and *Renilla* luciferase activities in the cells were measured at 48 h post-transfection, and the level of FLuc activity (normalized to that of *Renilla* luciferase in the respective sample) is shown as HAV IRES activity. (E) Organization of the HAV subgenomic replicon in which the majority of the structural protein-encoding region of the HM175/18f strain was replaced with the gene encoding FLuc. (F) Huh7.5.1 cells were transfected with the synthetic replicon RNA for 8 h, and then cultured in the absence or presence (at 2 or 10 μM) of loxapine succinate. At 72 h after transfection, the cells were harvested and FLuc activity was determined. The data are presented as the mean ± SD from three independent experiments. Statistical significance was determined using a two-tailed non-paired Student’s t test. * P < 0.05, ** P < 0.01 (vs. control).

We next investigated which step in the HAV life cycle was blocked by loxapine succinate. The HAV life cycle can be divided into three phases: (1) the early phase, which includes attachment, entry, and trafficking to the cytoplasm; (2) the replication phase, which includes translation and RNA genome replication; and (3) the late phase, which includes viral assembly and release. Cell culture-derived quasi-enveloped HAV (eHAV) has been shown to enter the cells via ALIX (ALG-2 interacting protein X)-dependent trafficking to lysosomes at approximately 6 hours post-infection (hpi) [11]; therefore, time-of-addition experiments were conducted, as shown schematically in Fig 2A. Time-of-addition experiments revealed that loxapine succinate shows no or slight inhibition of HAV under pretreatment or simultaneous exposure conditions. However, the compound showed significant inhibition of HAV under the post-treatment exposure condition (Fig 2A and 2B), suggesting that virus inhibition by exposure to loxapine succinate may reflect inhibition of steps after viral entry. To further assess the effect of loxapine succinate on internal ribosome entry site (IRES)-dependent translation of the HAV RNA, we used a bicistronic reporter plasmid, as shown in Fig 2C. Treatment of transfected cells with loxapine succinate did not result in attenuation of the activity of firefly luciferase (Fluc) expressed via HAV IRES-dependent translation (as normalized to the activity of *Renilla* luciferase expressed via cap-dependent translation). This result suggested that virus inhibition cannot be attributed to inhibition of HAV IRES-dependent translation.

We next evaluated the effect of loxapine succinate on viral RNA replication using an HAV subgenomic replicon that encodes the HAV nonstructural proteins essential for RNA replication, but not the structural proteins required for viral assembly (Fig 2E). As shown in Fig 2F, replication in this system was decreased by exposure to loxapine succinate, and this effect was dose dependent. These results suggested that loxapine’s inhibition of HAV propagation occurs via targeting of the viral RNA replication cycle.

### Mutations in the N-terminal domain of 2C abrogate the anti-HAV activity of loxapine succinate

Dopamine is a neurotransmitter that controls numerous physiologic functions in the brain and peripheral nervous system; effects of this compound are mediated by dopamine receptors (DRs) of the G-protein-coupled receptor (GPCR) superfamily. In humans, five DRs have been identified. Loxapine is known to primarily antagonize dopamine D2 receptors (DRD2s); the compound is used clinically as a therapeutic agent for the treatment of patients with schizophrenia. DRs are expressed primarily in brain tissue. In addition, recent studies have demonstrated the expression of DRs in immune cells [12]. However, it is not clear whether DR-encoding genes are transcribed in human hepatic tissues. RNA-seq analysis of Huh7.5.1 failed to detect any read alignment to DRD2-encoding transcripts (S1 Table). In addition, DRD2 expression (at either the protein or RNA level) is not detected in liver tissues or cells (The Human Protein Atlas; https://v17.proteinatlas.org). Therefore, we hypothesized that loxapine succinate inhibits HAV propagation in Huh7.5.1 cells independent of any effects on DRD2.

**Fig 3:**
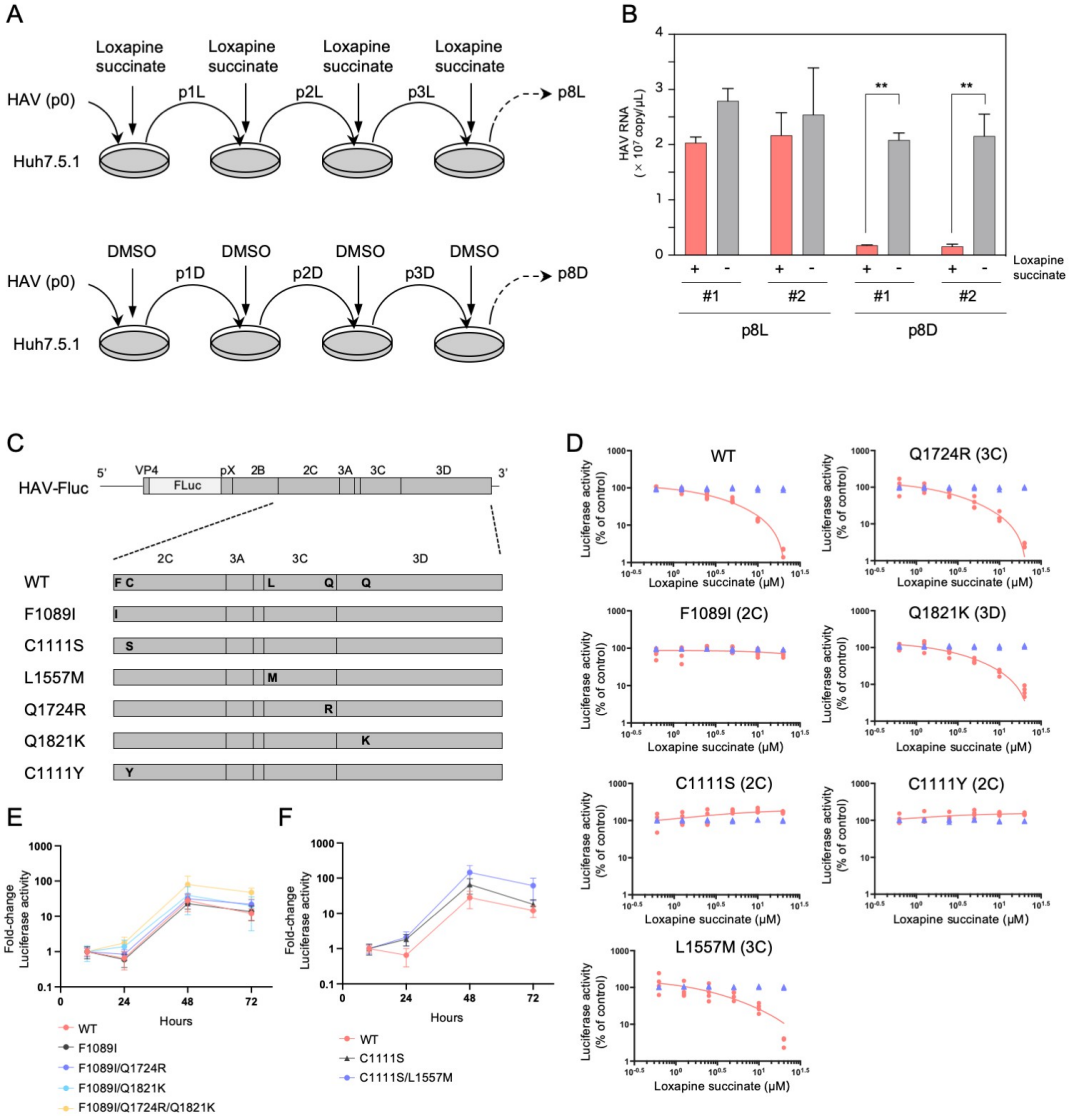
Loxapine-resistant mutations in HAV following blind passage. (A) Experimental procedure for blind passage of HAV. Huh7.5.1 cells were infected with HAV in the presence (10 μM) or absence of loxapine. Spent medium was collected and inoculated into naïve Huh7.5.1 cells. These procedures were repeated 8 times for two independent samples (#1 and #2). (B) Growth of HAV p8L and p8D (passaged in the presence and absence of loxapine, respectively) on Huh7.5.1 cells was assessed in the presence or absence of loxapine. Cells were infected with each HAV sample for 24 hours; at 7 days post-infection (dpi), spent medium was collected and subjected to RT-qPCR. Statistical significance was evaluated using a two-tailed non-paired Student’s t-test. ** P < 0.01 (vs. control). (C) Schematic representation of HAV subgenomic replicon structure. The identities and positions of amino acids altered by mutations in the HAV genome are shown. (D) Expression of reporter gene in HAV replicons. Huh7.5.1 cells were transfected with *in vitro*-synthesized RNAs for 4 h, and then cultured in medium containing the indicated concentrations of loxapine; Luc activity (red) and cell viability (blue) were determined at 3 days post-transfection. (E, F) Replication capacity of HAV subgenomic replicons encoding the 2C protein with (E) a F1089I substitution or (F) a C1111S substitution. *In vitro*-synthesized RNA was transfected into Huh7.5.1 cells, and the cells were harvested at 10, 24, 48, and 72 h post-transfection. The luciferase activity in the cell lysates was measured and expressed as the fold increase from the values at 10 h post-transfection (to correct for transfection efficiency). Independent assays were performed in quadruplicate, and the data are presented as the mean and standard deviation of relative light units (RLU).

To determine the mechanism(s) of loxapine inhibition of HAV replication, we isolated loxapine-resistant HAV. To this end, two-independent blind-passage experiments were conducted by sequentially inoculating culture supernatants from cells infected with HAV in the presence or absence of loxapine succinate (10 μM) (Fig 3A). Side-by-side infection analysis revealed that the two independent pools of virus subjected to 8 cycles of blind passaging in the presence of loxapine succinate (p8L) exhibited similar growth when cultured with cells in the presence or absence of loxapine succinate (Fig 3B). In contrast, viruses that had been blind passaged in the absence of loxapine succinate (p8D) remained susceptible to loxapine succinate. These results suggested that virus passaged in the presence of loxapine succinate had acquired resistance to the compound.

To identify the mutation(s) that confer resistance to loxapine, reverse transcription-polymerase chain reaction (RT-PCR) was used to amplify the genomes of passaged viruses as 2 fragments; the resulting nearly complete HAV genomes (lacking only part of the 5’-untranslated region (UTR; IRES-containing) and the 3’UTR region) were subjected to DNA sequence determination. Sequencing of the genome from two independent p8L isolates revealed that both harbored nonsynonymous mutations in the sequences encoding the 2C and 3C proteins, and that one clone (#1) contained a nonsynonymous mutation in the sequence encoding the 3D protein (Table 1). In contrast, no mutations were observed in two independent isolates from viruses subjected to 8 passages in the absence of loxapine (p8D). To determine the role of the mutations identified in the p8L HAV genomes, each of the individual mutations in the sequences encoding the 2C, 3C, and 3D proteins was introduced into a subgenomic replicon of HAV (Fig 3C). The resulting subgenomic clones, encoding 2C harboring an F1089I substitution (2C(F1089I)), 2C(C1111S), 3C(L1557M), 3C(Q1724R), or 3D(Q1821K) (along with a wild-type (WT) clone) were subjected to *in vitro* transcription; the resulting RNAs were used to transfect Huh7.5.1 cells. In addition, subgenomic clones encoding 2C harboring a C1111Y substitution (2C(C11111Y)) also were examined, given that tyrosine is detected (at high frequency) at residue 1111 of the 2C protein (see Discussion). The FLuc activities of replicons encoding 3C(L1557M), 3C(Q1724R), or 3D(Q1821K) were decreased by exposure to loxapine succinate, with the effects resembling those seen for the WT replicon. In contrast, the luciferase activities of HAV-FLuc encoding 2C(F1089I), 2C(C1111S), or 2C(C1111Y) demonstrated resistance to loxapine succinate (Fig 3D) compared to the WT replicon. Compared to WT, the replicon encoding 2C(F1089I) was not significantly altered in replication efficiency (Fig 3E). In addition, engineering of mutant replicons encoding 2C(F1089I) to additionally encode 3C(Q1724R) and/or 3D(Q1821K) mutant protein did not appear to compensate for the potential fitness cost. In contrast, a replicon encoding 2C(C1111S) showed nominally increased replication efficiency compared to WT, and a replicon encoding additional 3C(L1557M) mutation did not appear to alter replication efficiency compared to mutant replicon with 2C(C1111S) alone (Fig 3F). These results suggested that amino acid substitutions in the N-terminal domain of protein 2C confer resistance to loxapine, although we cannot completely exclude the possibility that other synonymous mutations in the sequences encoding pX or 3C contribute to viral resistance to this compound.

Given that the replication of a subgenomic replicon harboring mutations in the 2C-encoding sequences showed resistance to loxapine (Fig 3D), we conjectured that the effect of the compound on late stages of infection, such as assembly and egress, might be evaluated using a replicon containing such a resistance mutation. To this end, we used a *trans*-complemented HAV production system employing a subgenomic replicon in combination with a plasmid encoding HAV’s structural proteins (S2A Fig). When Huh7.5.1 cells were transfected with HAV-Luc (WT or F1089I) in the absence of co-transfection with the structural protein-encoding plasmid, the cell lysate of transfected cells (at Day 3) showed Luc activity (demonstrating that RNA replication was occurring) (S2B Fig). In contrast, no Luc signal was observed in cells that were infected with the supernatant derived from cells transfected with HAV-Luc (WT or F1089I) alone (S2C Fig), suggesting that no infectious HAV particles were generated in these transfected cells. For Huh7.5.1 cells transfected first with HAV-Luc (WT or F1089I) and then with the plasmid encoding VP4-pX, lysates exhibited Luc activity levels similar to those seen in the absence of the subsequent plasmid transfection. However, high Luc activity was seen in naïve cells following inoculation with the supernatant from cells co-transfected with HAV-Luc and the plasmid encoding VP4-pX. These results suggested that the cells co-transfected with HAV-Luc and the plasmid produced infectious *trans*-complemented HAV particles (HAVtcps). In these HAVtcps, the packaged genome corresponded to the subgenomic replicon; the supernatant containing these HAVtcps could in turn be used to transduce naïve cells. Loxapine succinate treatment of cells co-transfected with HAC-Luc (WT) and the plasmid resulted in the attenuation of Luc activity, both in the primary cell lysate and in subsequent HAVtcp-inoculated cells. In contrast, loxapine succinate treatment of cells co-transfected with HAV-Luc (F1089I) and the plasmid resulted in milder attenuation of Luc activity (compared to that seen with the WT replicon). Furthermore, loxapine succinate did not inhibit the Luc activity of cells inoculated with supernatant containing HAVtcps generated by cells co-transfected with HAV-Luc (F1089I) and the plasmid (S2B and S2C Fig). These results suggested that treatment with loxapine has no or minimal impact on the late stages of the HAV life cycle, such as assembly and egress.

**Table 1:** Summary of nucleotide and amino acid sequence differences between the parent virus and virus passaged in the presence of loxapine succinate (p8L).

HAV (p8L)	Mutation		Protein
	Nucleotide	Amino acid	
#1	T4011A/T	F1089F/I	2C
	T5912A/T	-	3C
	A5917A/G	Q1724Q/R	3C
	C6207A/C	Q1821Q/K	3D
#2	T3146C	-	pX
	T4077A	C1111S	2C
	C5415A/C	L1557L/M	3C

### Effect of loxapine treatment and 2C mutations on membrane reorganization mediated by 2C expression

Previous reports indicated that the expression of HAV 2C induces rearrangements of intracellular membranes [13]. To analyze the effect on membrane reorganization of loxapine treatment, as well as those of loxapine-resistance-conferring mutations in the N-terminal domain of protein 2C, we constructed plasmids encoding 2C proteins both with and without these mutations. Cells transfected with these plasmids were harvested after 48 h and examined by electron microscopy. As shown in S3 Fig, expression of HAV 2C (WT) induced coiled membranes, a state that has been designated as crystalloid endoplasmic reticulum (cER). cER also was observed in cells expressing HAV 2C with F1089I or C1111S mutations, as well as in 2C (WT)-expressing cells treated with loxapine. Therefore, the morphological rearrangement of membrane structure induced by 2C expression is not altered by exposure to loxapine, nor by the introduction (in the 2C protein) of N-terminal loxapine-resistance mutations.

### Characterization of the binding of loxapine to the HAV 2C protein

**Fig 4:**
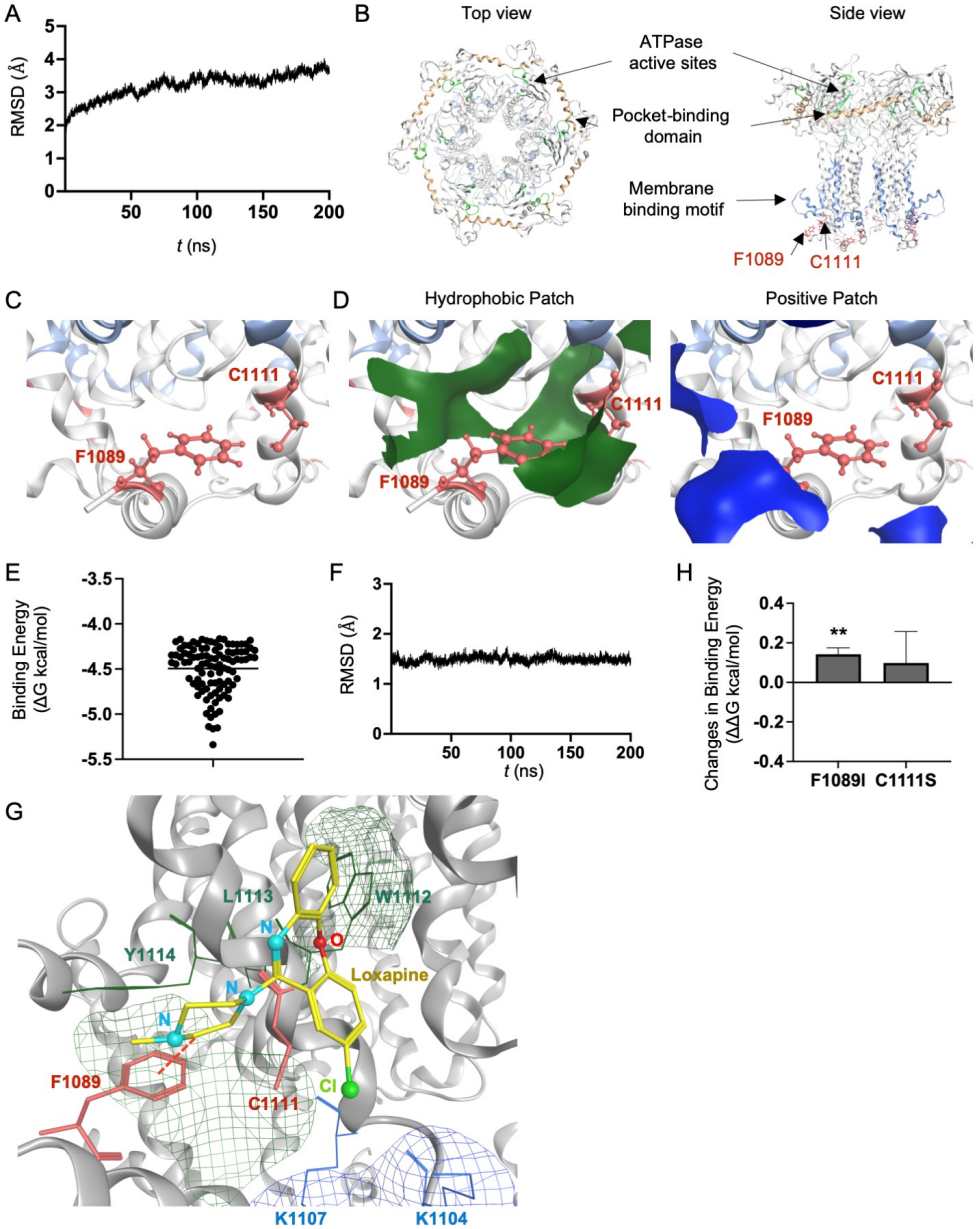
Characterization of possible binding of loxapine to the HAV 2C hexamer. (A) Root-mean-square deivations (RMSDs) between the initial model structure and the structures at the indicated time points during the molecular dynamics (MD) simulation of the 2C hexamer. (B) 2C hexamer structure at 200 ns of MD simulations. Red sticks show the side chains of the F1089 and C1111 amino acid residues. Orange, light green, and light blue portions indicate the pocket-binding domain [6], ATPase active sites [6] and membrane-binding motif [16], respectively. (C) Enlarged view of the region including the F1089 and C1111 amino acid residues. (D) Molecular patches relevant to hydrophobic and electrostatic interactions. Green portions indicate the hydrophobic patches that potentially are involved in the interactions between the hydrophobic moieties of molecules. Blue portions indicate the positively charged patches that potentially interact with negatively charged molecules. (E) Distribution of binding energies of the top-100 binding poses of the structure of the 2C-loxapine complex. Line indicates mean value. (F) RMSDs during the MD simulation of the 2C-loxapine complex. (G) Binding modes between 2C and loxapine. The two mutation sites (F1089 and C1111) are shown as red sticks. The red dotted lines show the arene interactions between loxapine and F1089. Blue, red, and green portions in the loxapine structure indicate nitrogen, oxygen, and chlorine atoms, respectively. The green and blue patches show the hydrophobic and positively charged regions, respectively. (H) Effects on the binding affinity of loxapine for the 2C hexamer. Changes in the binding affinity of loxapine were calculated using the structure of the 2C-loxapine complex obtained at 200 ns of MD simulations using the Protein Design application of MOE. Statistical significance was determined using a two-tailed non-paired Student’s t test. ** P <0.01 (vs. wild type (WT)).

*Picornavirus* 2C is a highly conserved multifunctional protein that is essential for various steps in the viral lifecycle [14]. HAV 2C has been predicted to comprise an N-terminal domain responsible for membrane binding [15]; a central ATPase domain essential for genome replication; and a C-terminal domain including a zinc-finger equivalent to the enterovirus 2C region (ZFER), as well as an amphipathic helix [6]. The 2C(F1089I) or 2C(C1111S) mutations associated with loxapine resistance are located in the N-terminal region of the protein, proximal to the membrane-binding site. To gain structural insights into the impact of such mutations in 2C on the interaction between 2C protein and loxapine, molecular dynamics (MD) simulations were conducted. We first constructed a model of the 2C hexamer and then subjected this model to MD simulations to delineate the dynamic behaviors of this biological macromolecule in response to the thermal motions of atoms and molecular collisions that would occur in solution. This *in silico* technique previously has been employed to characterize the physical properties of biomolecules under near-physiological conditions [16]. Structural dynamics during the simulations were evaluated using root-mean-square deviation (RMSD) between the initial model structure and the structures at given time points. The RMSD of the 2C hexamer achieved a near plateau immediately after the start of simulation (Fig 4A). The results suggested that the 2C hexamer structure reaches a state of thermodynamic equilibrium under solution conditions, as assessed by MD simulations. Next, the 2C structure at the 200-ns (plateaued) time point of the MD simulations was used to characterize the structural features of the F1089 or C1111 residues that were the sites of loxapine-resistance mutations. Both of these residues are located in the N-terminal domain of 2C, proximal to the membrane binding site [17]. The side chains of the F1089 and C1111 amino acid residues were exposed (Fig 4B and 4C). Notably, patch analysis of the three-dimensional structure of 2C showed that the F1089 and C1111 residues are located near hydrophobic and positively charged patches (Fig 4D).

We extended our analysis by conducting *in silico* simulations of docking between the 2C hexamer structure (at the 200-ns time point of the MD simulation) and loxapine, with the goal of clarifying the chemically, structurally, and thermodynamically appropriate binding site(s) of loxapine on 2C. We used the Dock tool in MOE (the Molecular Operating Environment in Fingerprints software; see Materials and methods) to identify the top-100 docking poses ranked based on the degrees of binding affinity and steric hindrance (Fig 4E). Next, we examined individual docking poses. Among the docking poses, the highest-docking-score (top-1) binding modes near F1089 and C1111 were seen the most frequently. To assess the stability of the 2C-loxapine complex, we ran an MD simulation using the top-1 binding mode. The RMSDs increased sharply after the onset of the MD simulations, achieving a near plateau after 10 ns (Fig 4F). Consequently, no major shift in the binding site was detected before or after the MD simulation. Together, these results suggested that the top-1 binding mode is thermodynamically stable under solution conditions.

To validate the modeling method used to characterize the HAV 2C-loxapine complex, we evaluated the binding affinity of loxapine for the EV-D68 2C hexamer, given that loxapine did not show antiviral activity against EV-D68 (S1 Fig). Specifically, we constructed a molecular model of the EV-D68 2C hexamer docked to loxapine using homology modeling, MD simulation, and *in silico* docking simulation, as described in Materials and Methods. Using the EV-D68 2C hexamer model in the equilibrium state under solution conditions, we conducted *in silico* simulation of docking between EV-D68 2C and loxapine (S4A Fig). The distribution of the free energy of binding (ΔG) showed that various docking poses were physiochemically possible. The range of values of the docking scores for the interaction between EV-D68 2C and loxapine was higher than that for the interaction between HAV 2C and loxapine. These results suggested that loxapine binds to the HAV 2C more readily than to EV-D68 2C. Next, to assess the stability of the EV-D68 2C-loxapine complex, we ran an MD simulation using the top-1 binding mode (S4B Fig). The RMSDs increased sharply after the onset of the MD simulations, achieving a near plateau after 100 ns (S4C Fig). Notably, following MD simulation, loxapine was not predicted to bind to the EV-D68 2C hexamer (S4D Fig). Together, these results suggested that loxapine has low affinity for the N-terminal domain of EV-D68 2C, in contrast to the compound’s affinity for HAV 2C. These *in silico* results were consistent with the results of the *in vitro* assays of the antiviral effect of loxapine on EV-D68 (S1A Fig).

In extension of the EV-D68 2C analysis, we further examined the molecular interactions between the HAV 2C hexamer and loxapine using the top-1 structures derived from the MD simulations (Fig 4G). In the modeled structures, the aromatic rings of loxapine were located proximal to the hydrophobic region of the 2C N-terminal region, including amino acid residues W1112, L1113, and Y1114. As a result, loxapine’s negatively charged chlorine atom was located near the positively charged region of the 2C N-terminal region, including amino acid residues K1104 and K1107.

Next, we assessed the effects on the HAV 2C-loxapine complex models of *in silico* site-directed mutagenesis of the F1089 and C1111 sites, specifically by focusing on MD simulations at 190–200 ns. The binding affinities of loxapine for 2C appeared to be decreased following the introduction (*in silico*) of either the F1089I or C1111S substitution (Fig 4H). These *in silico* results were consistent with the *in vitro* results showing that mutations in the sequences encoding the N-terminal region of 2C confer upon HAV the ability to replicate in the presence of loxapine succinate.

### Structure-activity relationship analysis of tricyclic compounds

**Fig 5:**
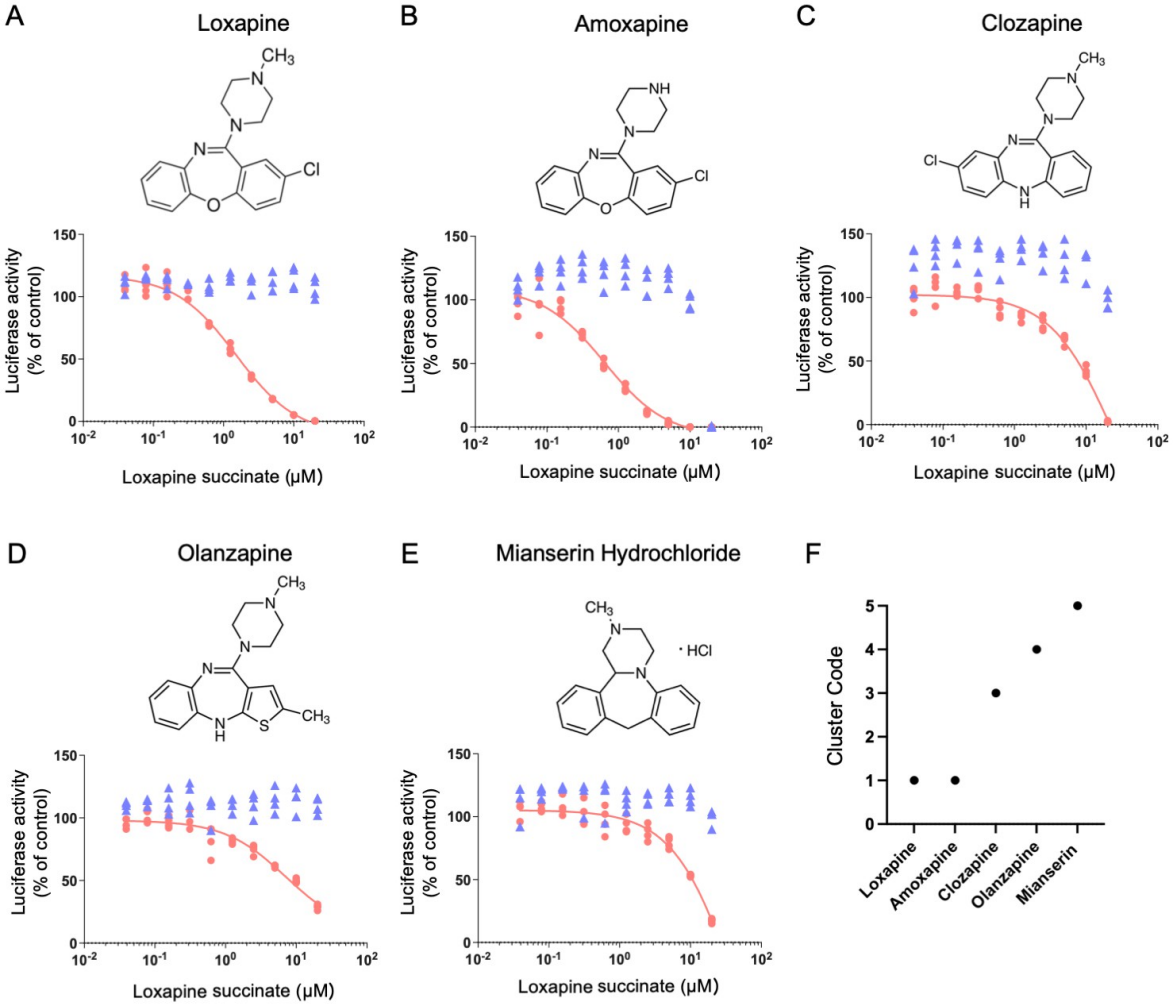
Effects on HAV replication of antipsychotic compounds that share the tricyclic structure of loxapine. Huh7.5.1 cells were infected with HAV/NLuc and cultured in growth medium containing loxapine succinate (A), amoxapine (B), clozapine (C), olanzapine (D), or mianserin hydrochloride (E) at the indicated concentrations. Nluc activity (red) and cell viability (blue) were determined at 72 hours post-infection (hpi). (F) Structural classification of antipsychotic compounds. Similarity of structural features among antipsychotic compounds, including loxapine, as assessed using the MOE Fingerprint Database Clustering application. The loxapine, amoxapine, clozapine, olanzapine, and mianserin structures were obtained from PubChem; the Compound Identification (CID) numbers of these compounds are 71399, 2170, 135398737, 135398745, and 4184 (respectively).

To further investigate potential antivirals with activity against HAV, we examined the effects of other antipsychotic compounds that contain tricyclic structures that resemble that of loxapine. Among such compounds, amoxapine, the *N*-demethylated derivative of loxapine, showed similar effects on HAV replication, although amoxapine exhibited severe cytotoxicity at 20 μM (Fig 5B). Another three related compounds showed weak activity against HAV (Fig 5C–5E). In confirmation of the structural similarity between amoxapine and loxapine, the two compounds were classified within a shared cluster when analyzed using the MOE Fingerprints software (Fig 5F). These results suggested that a tricyclic ring system with an alkyl amine substituent on the central ring, as well as the position of the chloride and oxygen, may be important for anti-HAV activity. These data also were consistent with the results of the *in silico* simulation of the HAV 2C-loxapine complex, which indicated that the inhibitor’s tricyclic ring is located proximal to amino acids 1111 and 1112 of the N-terminus of 2C (Fig 4G).

### *In vivo* antiviral efficacy of loxapine

**Fig 6:**
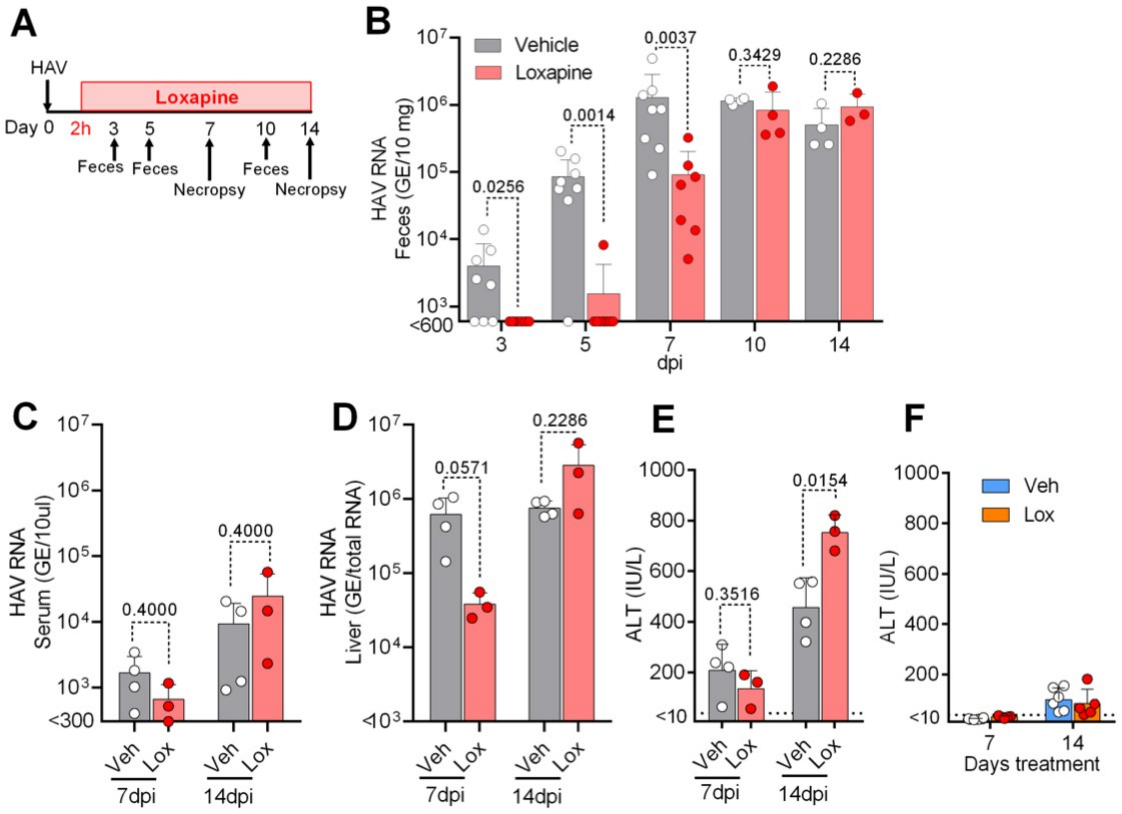
Efficacy of loxapine succinate treatment in HAV-infected female *Ifnar1*^-/-^ mice. (A) Experimental plan, showing groups of mice receiving postexposure prophylaxis by once-daily dosing with loxapine succinate (15 mg/kg) or vehicle starting 2 h after intravenous (i.v.) challenge with HAV at 1.0 × 10^6^ genome equivalents (GE) /mouse. Mice were monitored for serum levels of Ala aminotransferase (ALT) activity and fecal virus until necropsy at 14 days post-infection (dpi). Levels of (B) HAV RNA in feces (fecal shedding), (C) HAV RNA in serum, (D) HAV RNA in liver, and (E) serum ALT were measured during 14 days of treatment with loxapine or control (vehicle). (F) Serum ALT of uninfected mice during 14 days of dosing with loxapine or control (vehicle). The dotted horizontal line indicates the upper limit of normative ALT levels. The data are presented as mean ± SD (n = 4 or 8) in each group. Statistical analysis was performed by a two-tailed Mann-Whitney test or unpaired Student’s t test, and P values are provided in the figure. Data for the levels of HAV RNA in feces, liver, and serum, and of serum ALT in individual mice, are summarized in S2 Table.

As a final analysis, we investigated the effect of loxapine succinate on HAV replication in *Ifnar1*^*−/−*^ mice; given a lack of expression of the type I IFN receptor and a resulting deficiency in IFN signaling, these mice are permissive for HAV infection [9]. Groups of *Ifnar1*^*−/−*^ mice (n = 4 to 8) were inoculated intravenously with the liver homogenate from an *Ifnar1*^*−/−*^ mouse infected with HAV (Strain HM175; 12^th^ murine passage) at 1.0 × 10^6^ viral genome equivalents (GE) per animal. Starting at 2 hpi, and continuing for 14 days, animals received a once-daily intraperitoneal injection of vehicle or loxapine succinate (15 mg/kg) (Fig 6A). As expected, the control (vehicle-dosed) mice exhibited a rapid increase in the level of HAV RNA in the feces at 3 to 7 dpi. In comparison, the loxapine-dosed mice exhibited significant attenuation of the fecal level of HAV RNA through 7 dpi, although this effect of the compound was no longer observed at 10 and 14 dpi (Fig 6B). Characterization of the HAV sequences encoding the N-terminal region of 2C did not reveal any mutations (compared to the parent) in virus recovered (at 14 dpi) from the feces of HAV-infected mice administered loxapine (S5 Fig). Consistent with the observed effects on fecal levels of HAV RNA, the levels of HAV RNA in the serum and liver of the loxapine-dosed HAV-infected animals were attenuated compared to those seen in the control animals at 7 dpi, though not at 14 dpi (Fig 6C and 6D). Meanwhile, the serum levels of alanine aminotransferase (ALT; a biomarker of liver damage) in the loxapine-dosed infected mice were statistically indistinguishable from those in control (vehicle-dosed) infected mice at 7 dpi; in contrast, serum ALT levels in loxapine-dosed infected mice were significantly elevated at 14 dpi compared to those in the control infected mice (Fig 6E). Notably, ALT levels remained unaltered in mice dosed with loxapine in the absence of HAV infection (Fig 6F). Together, these data showed that loxapine succinate has an antiviral effect on HAV in an animal infection model, indicating that the compound delays the growth of this virus at the early stage of infection; however, these effects did not persist at later time points, as assessed by viral load and serum ALT levels.

## Discussion

Despite the successful development and deployment of a vaccine for hepatitis A, HAV remains a common cause of enterically transmitted hepatitis throughout the world, and is responsible for epidemics in both developing and developed countries. This challenge reflects, in part, the current lack of a HAV-specific antiviral treatment for infected patients. Clearly, a better understanding of the potential molecular targets of such antivirals is critical for the development of novel therapies. In an effort to identify small-molecule inhibitors of HAV infection, we conducted a chemical screen using HAV/NLuc; exposure to the highest-potency compound identified in this screen, loxapine succinate, was found to decrease HAV propagation by blocking viral RNA replication *in vitro*. This result is the first demonstration, to our knowledge, that loxapine succinate inhibits HAV propagation. Loxapine is a US FDA-approved first-generation antipsychotic medication that is used clinically, primarily in the treatment of patients with schizophrenia. The antipsychotic activity of loxapine is attributed to its antagonism of DRD2s. DRD2s are GPCRs that bind dopamine, a neurotransmitter that controls numerous physiologic functions in the brain and peripheral nervous system. While DRD2s classically are thought to be expressed in the nervous system, a recent study detected the additional presence of DRs in immune cells [12]. However, information on DRD2 expression in the human liver is limited. As part of the current study, we performed RNA-seq analysis of Huh7.5.1, a human hepatoma-derived cell line, but this analysis failed to detect any read alignment with transcripts encoding DRD2s (S1 Table). This result suggested that the effect of loxapine succinate on HAV replication occurs in a DRD2-independent manner.

To address the molecular mechanism of loxapine’s antiviral activity, we performed blind passaging of HAV in the presence of loxapine succinate to obtain resistant viruses; we expected that analysis of any resulting mutants would illuminate the molecular target of loxapine’s inhibition of HAV replication. The mutations conferring loxapine resistance mapped to sequences encoding the N-terminal membrane-binding domain of the HAV 2C protein. HAV 2C, which consists of 335 amino acids, is a multifunctional protein essential for viral replication. The structure of HAV 2C is predicted to comprise an N-terminal amphipathic α-helix domain responsible for membrane binding [15], a central ATPase domain essential for virus replication, and a C-terminal domain. The most conserved domain of HAV 2C is the central region, which shows homology to the ATPase domain of the superfamily 3 (SF3)-helicase family of ATPases associated with diverse cellular activities (AAA+) proteins. The C-terminal region of HAV 2C is functionally equivalent to the enterovirus 2C protein, although the zinc-finger domain of HAV 2C lacks the Cys-rich motif present in the enteroviral ZFER domain. The C-terminal helix of HAV 2C forms a hydrophobic pocket, mediating 2C-2C interactions; this domain is essential for self-oligomerization [6]. HAV 2C also has been shown to induce intracellular membrane rearrangement to provide a platform for assembly of the viral replication complex [13], and to recognize the 3’-UTR of negative-stranded viral RNA [18]. The 2C protein of picornaviruses is an attractive target for direct-acting antivirals (DAAs), given that picornavirus 2C is a highly conserved and functionally indispensable protein with multiple functions in the viral life cycle. In fact, the selective serotonin reuptake inhibitor (SSRI) fluoxetine has been shown to inhibit replication of human enteroviruses by targeting the 2C protein [19,20].

Data from time-of-addition experiments, as well as assays using a subgenomic replicon and *trans*-packaging experiments, suggested that loxapine acts as an inhibitor of viral replication, but not of the entry, translation, assembly, or release steps. In addition, subgenomic replicons containing mutations encoding 2C with F1089I or C1111S substitutions were able to replicate in the presence of loxapine. Therefore, we hypothesized that loxapine binds to 2C (WT), thereby attenuating the replication of HAV RNA. Our MD simulations confirmed that loxapine is capable of binding to the N-terminal region of the 2C protein, and predicted that both the F1089I and C1111S mutations (identified by blind passaging) would reduce the affinity of loxapine for 2C. It is unclear, however, how the binding of loxapine to the N-terminal region of 2C attenuates HAV RNA replication. One possibility is that loxapine’s binding to 2C impedes targeting of the protein to the cellular membrane, a process that may be essential for the intracellular formation of HAV replication complexes. However, we observed that the expression of HAV 2C (WT) induced the formation of cER, both in the presence and absence of loxapine.

According to the hepatitis virus database (https://hepjapandb-v2.nih.go.jp/hepatitisDB/program/top.cgi), a phenylalanine at amino acid residue 1089 is conserved among the 2C proteins encoded by HAV genotypes IA, IB, and IIA. In contrast, the presence of an isoleucine at this residue is conserved among the 2C proteins encoded by HAV genotypes IIIA and IIIB. On the other hand, a tyrosine is present at residue 1111 of the 2C protein in all but one of the known HAV genotypes. The sole exception is genotype IB, in which 34% of isolates harbor a cysteine at this residue. Notably, the HAV/NLuc strain, which was used here for initial screen, also encodes 2C with a C1111 residue. In contrast, no isolates have been reported to encode a 2C protein harboring a serine residue at this position. Taken together, these observations suggest that loxapine may exhibit activity against specific genotypes of HAV, an inference that will need to be tested in further research.

With regards to the amino acid encoded at residue 1111 of the HAV 2C protein, a subgenomic replicon derived from the HM175/18f strain encodes 2C with a Cys at position 1111. Notably, a mutant of this replicon with resistance to loxapine harbored a C1111Y substitution, as shown in Fig 3D. In contrast, all of the other HAV strains used in the present study encode the 2C protein with a Tyr at residue 1111, including Strain KRM031 and TKM005 (as shown in Fig 1F and 1H) and Strain HM175, used for murine passaging (as shown in Fig 6). Replication of these viruses was attenuated by exposure to loxapine succinate. We postulate that other amino acid residues in 2C may compensate for the sensitivity to loxapine (in strains other than HM175/18f) attributable to the Tyr at residue 1111 of 2C.

It is curious that loxapine succinate inhibits the propagation of HAV, but not that of EV-D68, despite the fact that both of these viruses belong to the family *Picornaviridae*. In addition, loxapine succinate inhibits the propagation of HCV, but not that of DV, despite the fact that both of these viruses belong to the family *Flaviviridae*. While we have not tested which step in the HCV life cycle is blocked by loxapine succinate, we conjecture that loxapine targets a specific HCV protein, as seen for HAV. These issues will need to be addressed in future research.

As a final assay, we evaluated the *in vivo* activity of loxapine using a murine model of HAV infection that recapitulates important aspects of the liver disease and viral replication kinetics seen clinically in patients infected with HAV. Following inoculation with HAV, *Ifnar1*^*-/-*^ mice (which have increased susceptibility to infection by this virus) become viremic, shedding virus in their feces; infected animals then develop acute hepatic inflammation (consistent with hepatocellular apoptosis) and exhibit elevated serum levels of ALT [9]. In this murine model of HAV infection, treatment with loxapine resulted in significant attenuation of fecal HAV RNA levels at 3–7 dpi; however, the attenuation of HAV RNA levels observed in feces, serum, and liver was no longer seen at 10 dpi. Indeed, ALT levels at 14 dpi were significantly elevated in loxapine-dosed HAV-infected mice compared to control (vehicle-treated) virus-infected animals. These *in vivo* results suggested that while loxapine delays the peak of viremia and fecal HAV RNA levels, this compound does not prevent HAV replication and pathogenesis in a murine model of hepatitis A.

It remains to be seen whether loxapine succinate suppresses HAV replication in humans. In the mouse model, treatment with loxapine significantly impaired HAV replication at early time points, but this effect did not persist at later time points. If loxapine does delay the onset of hepatitis A in humans, this compound may potentiate the effect of HAV vaccination when combined with the combination (loxapine succinate plus vaccination) for post-exposure treatment. However, a recent publication reported that oral administration of RG7834 (a small-molecule dihydroquinolizinone inhibitor of the terminal nucleotidyltransferases 4A and B (TENT4A/B)) in HAV-infected *Ifnar1*^*-/-*^ mice completely blocked viral infection and provided strong attenuation of hepatitis-induced elevation of serum ALT levels [21]. The potency observed for RG7834 implies that the anti-HAV activity of loxapine may not be sufficient for clinical use; further compound structure development, as well as further animal experimental data, likely will be needed before loxapine-class compounds are tested as anti-HAV treatments in clinical trials.

In conclusion, these studies revealed that loxapine succinate possesses anti-HAV activity, both *in vitro* and *in vivo*. To our knowledge, this work is the first to show that loxapine succinate exhibits antiviral activity against HAV replication, including the suggestion that this effect may be independent of DRD2. Given that passive and active immunization regimens are used widely for post-exposure prophylaxis of hepatitis A, we hypothesize that administration of loxapine succinate may enhance the effect of such prophylactic treatments by delaying initial HAV replication.

## Materials and methods

### Ethics statement

All animal experiments were approved by the Animal Care and Use Committee of the National Institute of Infectious Diseases (approval no. 122002) and carried out in accordance with the approved guidelines.

### Cell culture

Cells of the human hepatoma-derived Huh7.5.1 line, human embryonic kidney 293T line and the human rhabdomyosarcoma RD-A line were maintained in Dulbecco’s Modified Eagle Medium (DMEM) supplemented with nonessential amino acids, penicillin at 100 U/mL, streptomycin at 100 μg/mL, and 10% fetal bovine serum (FBS). All cultures were grown at 37 °C in a 5% CO_2_ incubator.

### Gene expression profile of Huh7.5.1 cells

Total RNA was extracted from Huh7.5.1 cells. The RNA-seq analysis was performed at Macrogen (Soeul, Republic of Korea) by obtaining 100-bp paired-end reads (approximately 40 million reads total) using Illumina (San Diego, CA, USA) NovaSeq 6000 machines. Mapping was conducted by Spliced Transcript Alignment to a Reference (STAR). The resulting data are summarized in Supporting Information (S1 Table).

### Plasmids

To generate the bicistronic reporter plasmid pRL-HAV18f-L, the HAV IRES (nucleotides 1–746 of the HAV HM175/18f genome) [22] and the firefly luciferase-encoding gene from pGL3-Basic (Promega) were inserted into the XbaI site of pRL-CMV (Promega).

The pHAV-Luc plasmid was described previously [7]. pHAV-Luc derivatives harboring mutations in the sequences encoding 2C (for F1089I, C1111S or C1111Y substitutions), 3C (for L1557M and Q1724R substitutions) or 3D (for Q1821K substitution) were constructed by oligonucleotide-directed mutagenesis.

To generate the plasmid encoding the VP4-pX segment of the HAV polyprotein from the genome of Strain HM175/18f, the corresponding DNA sequences were inserted into the pCAGGS vector.

### DNA transfection

293T cell monolayers were transfected with plasmid DNA using the Lipofectamine 3000 (ThermoFisher Scientific) according to the manufacturer’s instructions.

### Virus

The HAV HM175/18f strain and the HM175/18f-NLuc reporter virus were described previously [7], as were the HAV KRM031 and HAV TKM005 strains [23] and the D1/Hu/Saitama/NIID100/2014 strain of Dengue virus serotype 1 (DV1) [24]. All HAV strains were propagated using Huh7.5.1 cells. The Fermon strain of EV-D68 was obtained from ATCC (Manassas, VA, USA). The cell culture-produced hepatitis C virus (HCVcc) used in the present study was derived from the JFH-1 strain by the introduction of adaptive mutations in the sequences encoding E2 (generating an N417S substitution), p7 (generating an N765D substitution), and NS2 (generating a Q1012R substitution), as described previously [25].

### Luciferase assay and cell titer assay

After inoculation, the Huh7.5.1 cells were washed once with phosphate-buffered saline (PBS) and lysed with Passive Lysis Buffer (Promega). Luciferase activities in the lysates were determined using the Nano-Glo Luciferase Assay System (Promega). Cell viability was analyzed using the Cell Titer-Glo Luminescent Cell Viability Assay (Promega). For assays of cells harboring the subgenomic replicons, firefly luciferase (FLuc) activity was determined using the Luciferase Assay System (Promega).

### *In vitro* RNA transcription to generate the subgenomic replicons

Plasmids used for the generation of HAV subgenomic replicons were linearized by digestion with XmaI, and the resulting template DNAs were subjected to *in vitro* transcription using the mMESSAGE mMACHINE T7 Transcription Kit (ThermoFisher Scientific, Waltham, MA, USA). The yield and integrity of transcripts were assessed by gel electrophoresis under nondenaturing conditions, and aliquots of the transcription reaction products then were used for transfection, without additional purification.

### Transmission electron microscopy

293T cells were transfected with 2C-encoding plasmids as described above. At 2 days post-transfection, cells were fixed with 2% paraformaldehyde and 2.5% glutaraldehyde in 30 mM HEPES (4-(2-hydroxyethyl)-1-piperazineethanesulfonic acid) buffer (pH 7.4). The samples then were post-fixed in a 1% osmium fixation solution and embedded in Spurr resin. Thin sections were mounted on copper grids and post-stained with saturated uranyl acetate and lead citrate. Specimens were observed using an HT7700 transmission electron microscope (Hitachi High Technologies, Tokyo, Japan).

### RNA transfection

Synthetic replicon RNAs were transfected into Huh7.5.1 monolayers using Lipofectamine 3000 (ThermoFisher Scientific) according to the manufacturer’s suggested protocol.

### RNA extraction and reverse transcription-quantitative polymerase chain reaction (RT–qPCR)

RNA was extracted from serum and fecal samples using the QIAamp Viral RNA Isolation Kit (Qiagen, Valencia, CA) according to the manufacturer’s protocol. RNA was extracted from tissues using the TRIzol reagent (Invitrogen Life Technologies, Carlsbad, CA) according to the manufacturer’s protocol. RNA concentrations were measured using a NanoDrop device (ThermoFisher Scientific). Detection of HAV genome RNA was performed using one-step RT–qPCR analysis (TaqMan Fast Virus 1-step Master Mix: ThermoFisher Scientific) on a QuantStudio 3 real-time PCR system (ThermoFisher Scientific). HAV RNA levels were determined by reference to a standard curve generated with synthetic HAV RNA. Reactions were performed using primers targeting sequences in the 5’-UTR RNA segment of the genome; the primer sequences were as follows: AGGGTAACAGCGGCGGATAT and ACAGCCCTGACARTCAATYCMCT (where R is purine (A + G), Y is pyrimidine (C + T), and M is A + C). The FAM (6-carboxyfluorescein)/TAMRA (6-carboxytetramethylrhodamine) probe had the sequence AGACAAAAACCATTCAACRCCGRAGGAC [26]. Dengue virus (DV) and HCV RNA levels were quantified using specific primer pairs that targeted the RNA genomes of the respective viruses, as described previously [27] [28].

### EV-D68 titration

RD-A cells were plated at 1E+05 cells/well in 96-well, flat-bottom cell culture plates. After 24 h, serial 10-fold dilutions of EV-D68 were added to the appropriate wells and the plates were incubated at 37 °C for 6 days. All wells were examined for signs of cytopathic effects; 50% tissue culture infectious dose (TCID_50_) values were calculated using the Spearman & Kärber algorithm.

### Reagents

The Library of Pharmacologically Active Compounds (LOPAC^1280^) and ribavirin were obtained from Sigma-Aldrich (St. Louis, MO). Cyclosporin A was obtained from LKT Laboratories (St. Paul, MN). Pirodavir was a kind gift of Dr. Y. Nishimura [29].

### Molecular modeling of the HAV 2C hexamer

A three-dimensional model of the HAV 2C hexamer was constructed in the AlphaFold 2 program [30] using the reported amino acid sequence of the protein encoded by HAV HM175/18f strain [7]. The resulting 2C model exhibited a per-residue confidence score (pLDDT) of 89.0, which is in the “Confident” range [30]. The resulting model was optimized via energy minimization using the Molecular Operating Environment (MOE) (Chemical Computing Group, Montreal, Quebec, Canada) and an Amber 10: Extended Hückel Theory (EHT) force field implemented in MOE, which combines the Amber 10- and EHT-bonded parameters for large-scale energy minimization [31]. The 2C hexamer model was subjected to MD simulations as described previously for viral proteins [32–34]. Briefly, the simulations were performed using the pmemd.cuda.MPI module in the Amber 16 program [35] with the ff14SB force field for protein simulation [36]. The 2C hexamer was solvated in a truncated octahedral box of TIP3P-model water molecules with a distance of at least 9 Å around the 2C model [37]. A non-bonded cut-off of 10 Å was employed. Bond lengths involving hydrogen were constrained with SHAKE, a constraint algorithm that satisfies Newtonian motion [38]. The trajectory data of all MD simulations were collected at 2 fs intervals. After heating calculations were performed for 20 ps, at up to 310 K, using the NVT ensemble, simulations were executed using the NPT ensemble at 1 atm and 310 K, in 150 mM NaCl, for a total of 200 ns. The trajectory files during MD simulations were used to calculate RMSDs. RMSDs between the heavy atoms of the initial complex structure and the structure at given time points during the MD simulation were calculated to monitor the overall structural changes, as described previously [33,34,39]. RMSD values were calculated using the cpptraj module in AmberTools 16, a trajectory analysis tool [35].

### Molecular patch analysis

The interaction-prone areas on the 2C hexamer were estimated using the “Protein Patch Analyzer” tool in MOE, as described previously [39–42]. A hexamer model of the 2C protein after 200 ns of MD simulation was used for the patch analyses. Briefly, the Protein Patch Analyzer tool was applied to search for the positively charged patches (defined areas of at least 40 Å^2^) that potentially interacted with negatively charged molecules. The tool also was employed to search for the hydrophobic patches (defined as areas of at least 50 Å^2^) that potentially interacted with the hydrophobic moieties of molecules.

### Docking simulation of the HAV 2C hexamer and loxapine

Physiochemically and thermodynamically possible binding modes of loxapine to the 2C hexamer model were assessed using the Dock application of MOE, as described previously [33, 43]. The loxapine structure was obtained from PubChem (CID Number 71399). The structure of the 2C N-terminal region, including the F1089 and C1111 amino acid residues, and that of loxapine were defined as a receptor and ligand, respectively. Subsequently, possible docking poses between the receptor and ligand were evaluated by a comprehensive search under conditions that yielded the top-100 docking poses. A 2C model after 200 ns of MD simulation was used for the docking simulation. Binding energies of loxapine to 2C were calculated with the Dock tool using individual docking poses.

### MD simulation of the HAV 2C hexamer docked to loxapine

The model of the 2C-loxapine complex that was obtained by *in silico* docking simulation was subjected to MD simulation. Briefly, MD simulations were performed using the pmemd.cuda.MPI module in the Amber 16 program package with the ff14SB force field for simulation of protein, the gaff2 force field for simulation of organic molecules [44], and the TIP3P water model for simulation of aqueous solutions. A non-bonded cut-off value of 10 Å was used. Bond lengths involving hydrogen were constrained with SHAKE. The time step for all MD simulations was set to 2 fs. After heating calculations were performed for 20 ps up to 310 K using the NVT ensemble, simulations were executed for 200 ns in 150 mM NaCl using the NPT ensemble at 1 atm and 310 K.

### *In silico* site-directed mutagenesis of the HAV 2C-loxapine complex

Structures of the HAV 2C-loxapine complex (corresponding to the interval from 190 to 200 ns in the MD simulations) were used for *in silico* site-directed mutagenesis of the 2C protein. Modeling the 2C mutants (F1089I and C1111S) and calculation of mutation-associated changes in loxapine’s binding affinity for 2C were performed using the Protein Design application of MOE, along with the MM/GBVI program, as described previously [34,45,46]. The results were expressed as the change in the free energy of binding (ΔΔG).

### MD simulation of the EV-D68 2C hexamer docked to loxapine

A three-dimensional model of the EV-D68 2C hexamer model docked to loxapine was constructed using the modeling method employed for the HAV 2C-loxapine complex, as described above, with modification for the modeling of the EV-D68 2C hexamer. Briefly, the EV-D68 2C monomer was constructed in the AlphaFold 2 program using the reported amino acid sequence of the protein encoded by the EV-D68 Fermon strain (GenBank Accession No. YP_009508947) [47]. The EV-D68 2C hexamer model was constructed by homology modeling, and the HAV 2C hexamer model was used as the modeling template. The resulting EV-D68 2C hexamer model was subjected to MD simulation and *in silico* docking simulation with loxapine under the same conditions as those employed for the HAV 2C hexamer model, as described above.

### Mice

Animals were bred and housed at the National Institute of Infectious Diseases, Japan, in accordance with the policies and guidelines of the facility’s Institutional Animal Care and Use Committee. *Ifnar1*^*-/-*^ mice were provided by S. Morikawa of the National Institute of Infectious Diseases, Japan [48].

### Hepatitis A virus (HAV) infectious challenge

A homogenate of liver from *Ifnar1*^*-/-*^ mice infected with a 11th murine passage of HAV Strain HM175 virus was used to infect the mice of the present study. To generate the liver inoculum, the liver was recovered and homogenized in PBS; the homogenate then was cleared by centrifugation at 10,000 × g for 30 min at 4 °C, and aliquots of the resulting supernatant were stored frozen at −80 °C. The HAV RNA content (in genome equivalents; GE) of the cleared homogenate was quantified by real-time RT-qPCR as described above.

For the *in vivo* challenge experiments, female *Ifnar1*^*-/-*^ mice (6 to 10 weeks of age) were infected by intravenous injection with a volume of cleared liver homogenate corresponding to 1.0 × 10^6^ GE HAV RNA per mouse. Starting at 2 hpi, and continuing once daily for up to 14 days, animals (in groups of n = 8) were administered loxapine succinate at 15 mg/kg (or an equivalent volume of vehicle) by intraperitoneal injection. Animals were maintained on study for up to 14 dpi, during which fecal pellets and serum samples were collected at regular intervals. Following euthanasia of 4 mice/group on Days 7 and 14, tissues were harvested and stored in RNAlater (ThermoFisher Scientific) pending analysis.

### Alanine aminotransferase (ALT) assay

Serum levels of ALT were measured using the Fuji Dri-Chem Slide GPT/ALT-PIII kit (Fujifilm, Tokyo, Japan) according to the manufacturer’s protocol.

### Statistical analysis

Statistical tests were carried out using Prism (v. 6; GraphPad Software, La Jolla, CA, USA). Unless otherwise noted, comparisons between groups were conducted using a non-paired Student’s t-test or nonparametric Mann-Whitney test (for comparisons of data from two groups) or by One-way or Two-way Analysis of Variance (ANOVA). All analyses were performed as two-tailed tests; p <0.05 was considered statistically significant. Details of specific statistical tests and experimental design are provided in the relevant figure legends.

## Supporting information

S1 Fig Antiviral effect of loxapine succinate on the propagation of EV-D68, dengue virus (DV), and hepatitis C virus (HCV). (A) Huh7.5.1 cells were infected with EV-D68 at a multiplicity of infection (MOI) of 0.1; at 4 hours post-infection (hpi), cells were washed to remove extracellular virus and then cultured for 2 days in the absence or presence of loxapine succinate (10 μM) or pirodavir (13 μM), a known picornavirus inhibitor. Spent medium from the infected cells was titrated using the 50% tissue culture infectious dose (TCID_50_) assay. (B) Huh7.5.1 cells were infected with DV type 1 at an MOI of 0.01; at 4 hpi, cells were washed to remove extracellular virus and then cultured for 3 days in the absence or presence of loxapine succinate (10 μM) or ribavirin (100 μM), a known DV inhibitor. Extracellular DV RNA in the spent medium was quantified by RT-qPCR. (C) Huh7.5.1 cells were infected with HCV at an MOI of 0.05; at 4 hpi, cells were washed to remove extracellular virus and then cultured for 5 days in the absence or presence of loxapine succinate (10 μM) or cyclosporin A (5 μM), a known HCV inhibitor. Extracellular HCV RNA in the spent medium was quantified by RT-qPCR. Statistical significance was evaluated using a two-tailed non-paired Student’s t-test. ** P < 0.01 (vs. control). DMSO, dimethyl sulfoxide (vehicle control).
(TIF)

S2 Fig Effect of loxapine treatment on HAV assembly/release steps. Huh7.5.1 cells were transfected with subgenomic replicon RNA with or without mutation, followed by transfection with the VP4-pX-encoding plasmid (S2A Fig). Culture medium was replaced with fresh medium with or without loxapine (10 μM) at 6 hours post-plasmid transfection. At 3 days after transfection (with the replicon RNA), the spent medium (supernatant; sup) from the transfected cells was collected, diluted and used to inoculate naïve Huh7.5.1 cell monolayers. Luciferase activity of cells also was determined (S2B Fig). Luciferase activity of sup-inoculated cells subsequently was determined at 3 days post-infection (dpi) (S2C Fig). Statistical significance was evaluated using a two-tailed non-paired Student’s t-test. * P < 0.05, ** P < 0.01.
(TIF)

S3 Fig Morphological effects of expression of HAV 2C in 293T cells. Transmission electron micrographs (TEM) of cells transfected with the following: empty vector (control cells); plasmid encoding HAV 2C (WT); plasmid encoding HAV 2C (WT) and grown in the presence of loxapine succinate (10 μM) from 6 hr to 48 hr after transfection; plasmid encoding HAV 2C (F1089I); or plasmid encoding HAV 2C (C1111S). Crystalloid endoplasmic reticulum (cER) structures are indicated by pink circles.
(TIF)

S4 Fig Characterization of possible binding of loxapine to the EV-D68 2C hexamer. (A) Distribution of binding energies of the top-100 binding poses of the structure of the EV-D68 2C-loxapine complex. Line indicates mean value. (B) Binding mode between EV-D68 2C and loxapine in the top-1 model of *in silico* docking simulation. Orange, light green, and light blue portions indicate the pocket-binding domain, ATPase active sites, and membrane-binding motif, respectively. (C) Root-mean-square deviations (RMSDs) during the molecular dynamics (MD) simulations of the EV-D68 2C-loxapine complex. (D) Binding mode between EV-D68 2C and loxapine at 200 ns of MD simulation.
(TIF)

S5 Fig Electropherograms showing the sequences (on Day 14) of the inoculated virus and of HAV RNA extracted from feces of mice administered once daily with 15 mg/kg loxapine. Viral RNA from the inoculated HAV, and from HAV recovered on Day 14 via fecal shedding (from three independent mice) following 14 days of once-daily administration of 15 mg/kg loxapine, was extracted, reverse transcribed, and amplified by PCR. The complete 2C-encoding region (and flanking sequences) of the amplified cDNA was sequenced. The nucleotide sequences of the complete 2C-encoding region (and the partial 2B-encoding region), along with the predicted amino acid sequences, are indicated above the electropherograms.
(TIF)

S1 Table Supporting information tables. (XLSX)

S2 Table Supporting information tables. (XLSX)
