# Supplementary figures and images for: Loxapine inhibits replication of hepatitis A virus in vitro and in vivo by targeting viral protein 2C
Source: PLoS Pathog. 2024 Mar 13;20(3):e1012091. doi: 10.1371/journal.ppat.1012091 (PMC10962851; doi:10.1371/journal.ppat.1012091)

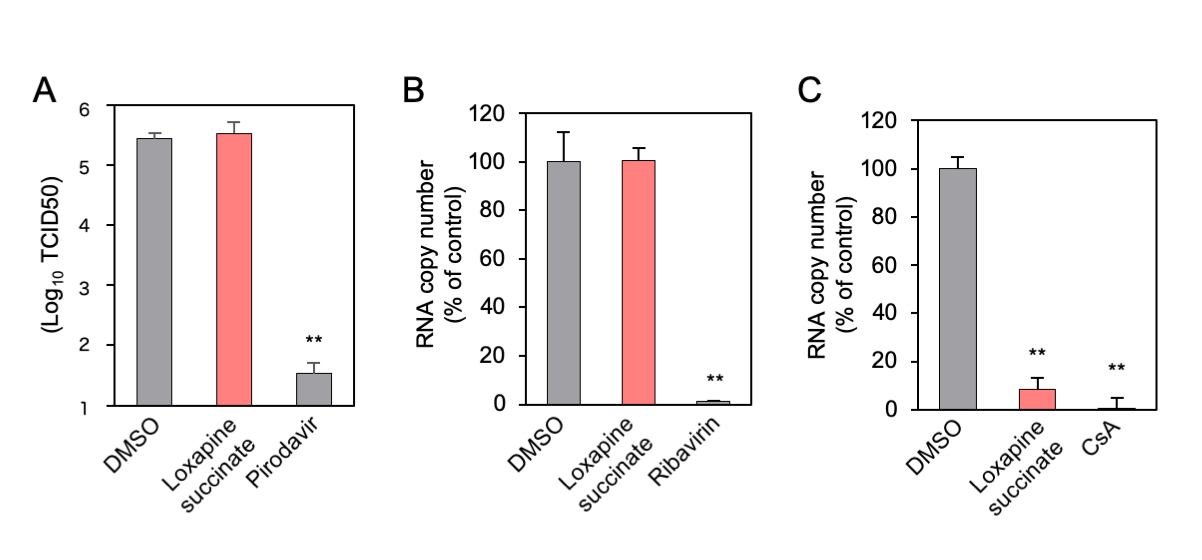

Supplement: S1 Fig — (A) Huh7.5.1 cells were infected with EV-D68 at a multiplicity of infection (MOI) of 0.1; at 4 hours post-infection (hpi), cells were washed to remove extracellular virus and then cultured for 2 days in the absence or presence of loxapine succinate (10 μM) or pirodavir (13 μM), a known picornavirus inhibitor. Spent medium from the infected cells was titrated using the 50% tissue culture infectious dose (TCID50) assay. (B) Huh7.5.1 cells were infected with DV type 1 at an MOI of 0.01; at 4 hpi, cells were washed to remove extracellular virus and then cultured for 3 days in the absence or presence of loxapine succinate (10 μM) or ribavirin (100 μM), a known DV inhibitor. Extracellular DV RNA in the spent medium was quantified by RT-qPCR. (C) Huh7.5.1 cells were infected with HCV at an MOI of 0.05; at 4 hpi, cells were washed to remove extracellular virus and then cultured for 5 days in the absence or presence of loxapine succinate (10 μM) or cyclosporin A (5 μM), a known HCV inhibitor. Extracellular HCV RNA in the spent medium was quantified by RT-qPCR. Statistical significance was evaluated using a two-tailed non-paired Student’s t-test. ** P < 0.01 (vs. control). DMSO, dimethyl sulfoxide (vehicle control). (TIF) [file ppat.1012091.s001.tif]

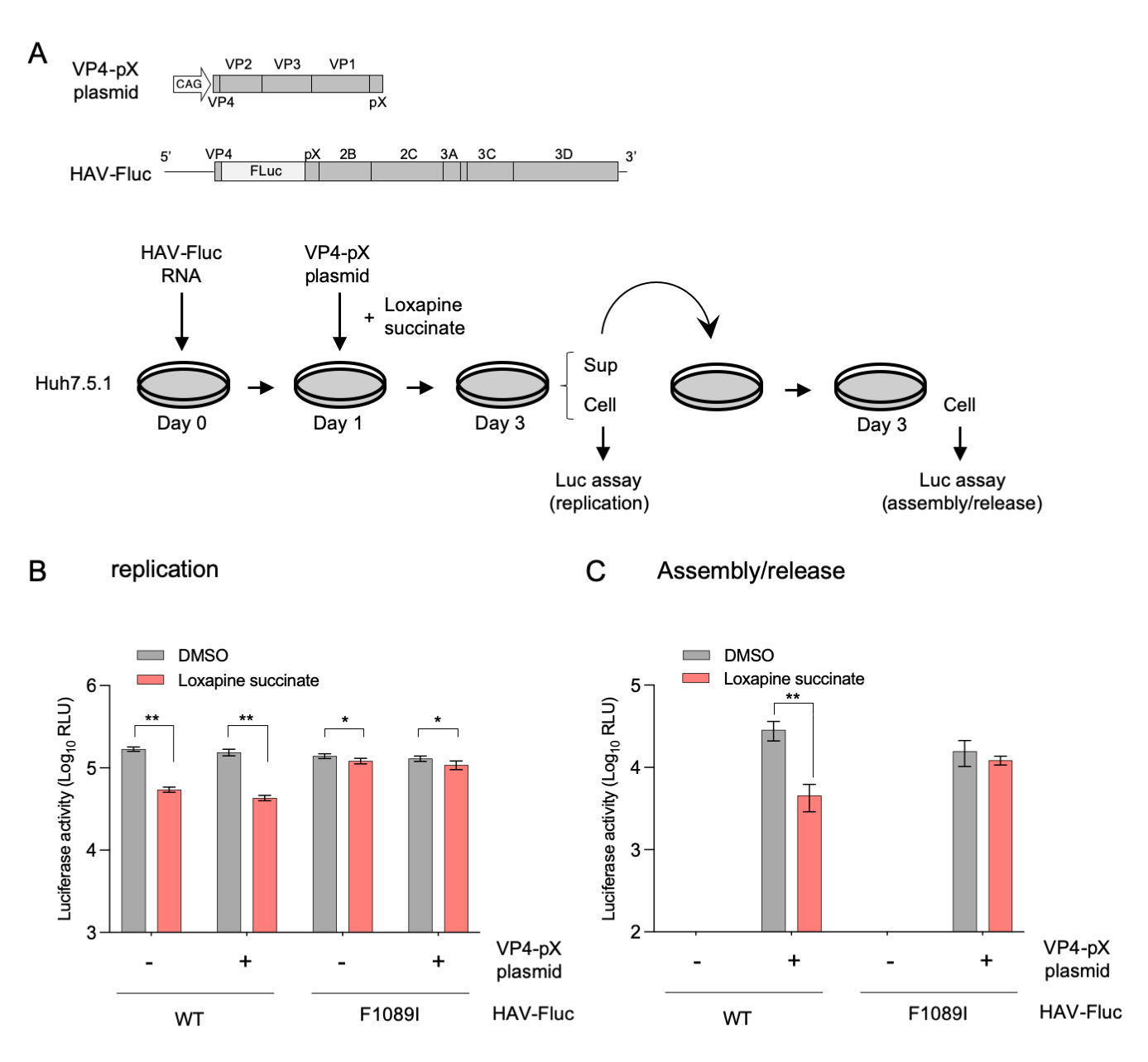

Supplement: S2 Fig — Huh7.5.1 cells were transfected with subgenomic replicon RNA with or without mutation, followed by transfection with the VP4-pX-encoding plasmid (S2A Fig). Culture medium was replaced with fresh medium with or without loxapine (10 μM) at 6 hours post-plasmid transfection. At 3 days after transfection (with the replicon RNA), the spent medium (supernatant; sup) from the transfected cells was collected, diluted and used to inoculate naïve Huh7.5.1 cell monolayers. Luciferase activity of cells also was determined (S2B Fig). Luciferase activity of sup-inoculated cells subsequently was determined at 3 days post-infection (dpi) (S2C Fig). Statistical significance was evaluated using a two-tailed non-paired Student’s t-test. * P < 0.05, ** P < 0.01. (TIF) [file ppat.1012091.s002.tif]

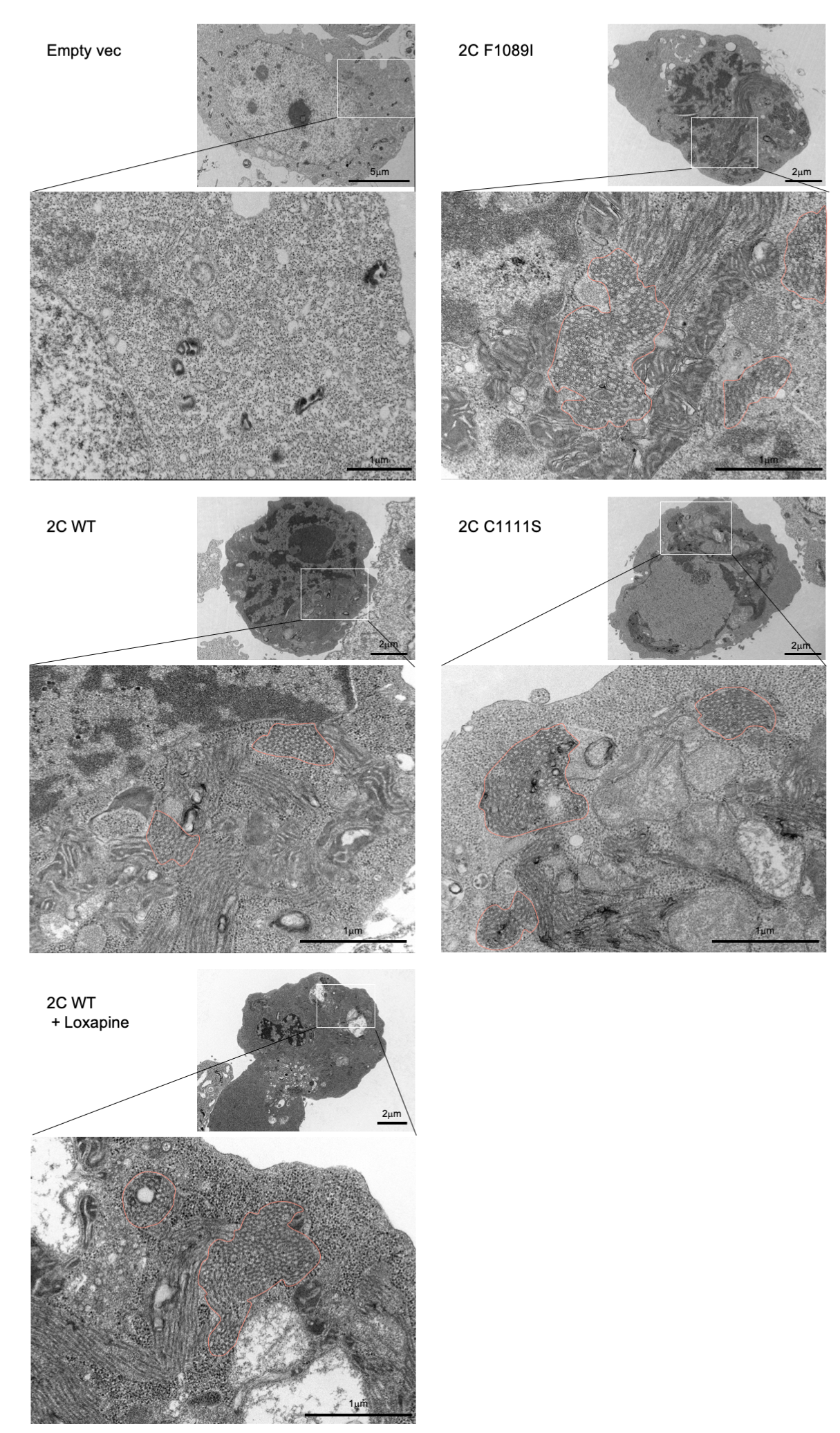

Supplement: S3 Fig — Transmission electron micrographs (TEM) of cells transfected with the following: empty vector (control cells); plasmid encoding HAV 2C (WT); plasmid encoding HAV 2C (WT) and grown in the presence of loxapine succinate (10 μM) from 6 hr to 48 hr after transfection; plasmid encoding HAV 2C (F1089I); or plasmid encoding HAV 2C (C1111S). Crystalloid endoplasmic reticulum (cER) structures are indicated by pink circles. (TIF) [file ppat.1012091.s003.tif]

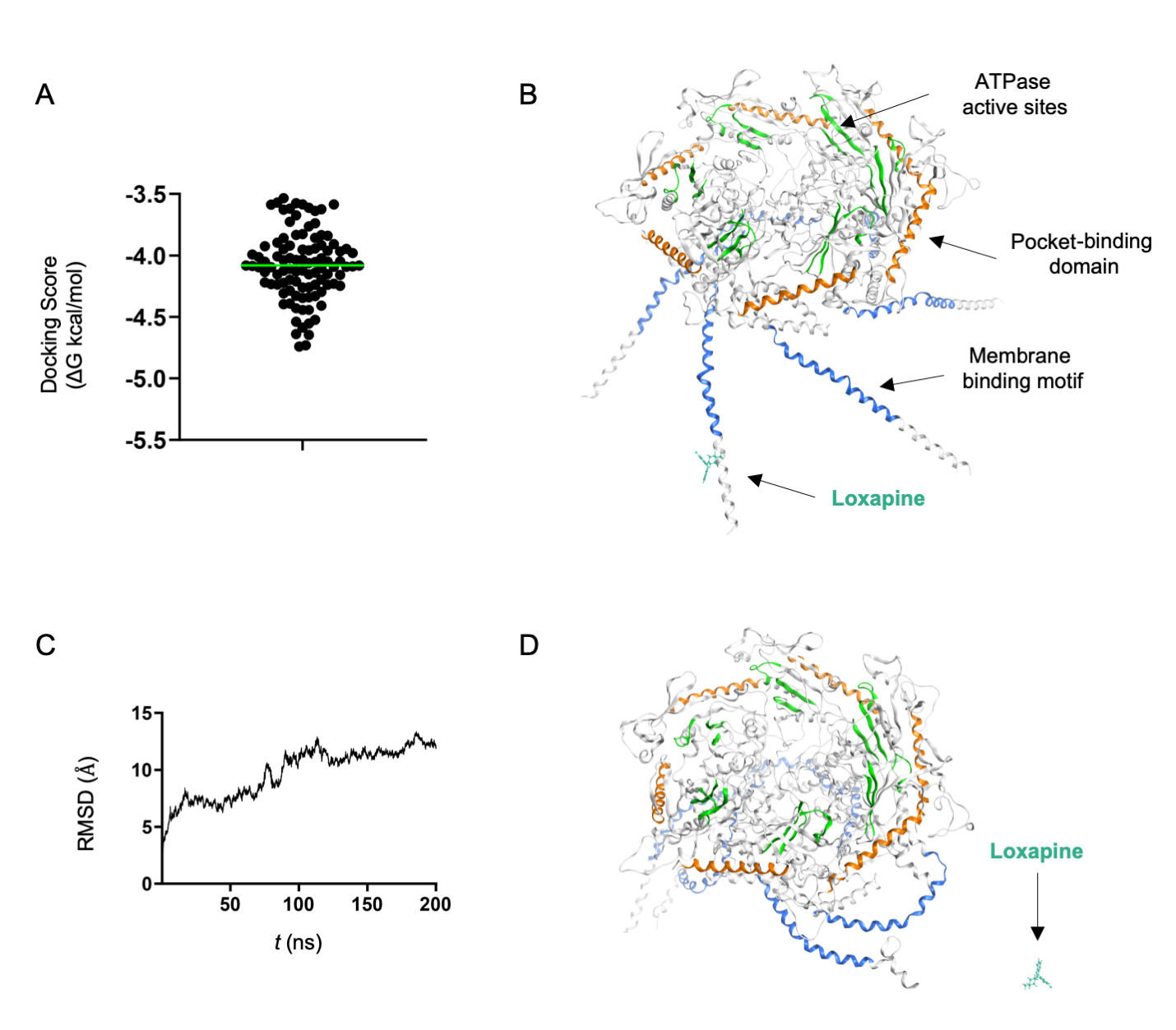

Supplement: S4 Fig — (A) Distribution of binding energies of the top-100 binding poses of the structure of the EV-D68 2C-loxapine complex. Line indicates mean value. (B) Binding mode between EV-D68 2C and loxapine in the top-1 model of in silico docking simulation. Orange, light green, and light blue portions indicate the pocket-binding domain, ATPase active sites, and membrane-binding motif, respectively. (C) Root-mean-square deviations (RMSDs) during the molecular dynamics (MD) simulations of the EV-D68 2C-loxapine complex. (D) Binding mode between EV-D68 2C and loxapine at 200 ns of MD simulation. (TIF) [file ppat.1012091.s004.tif]

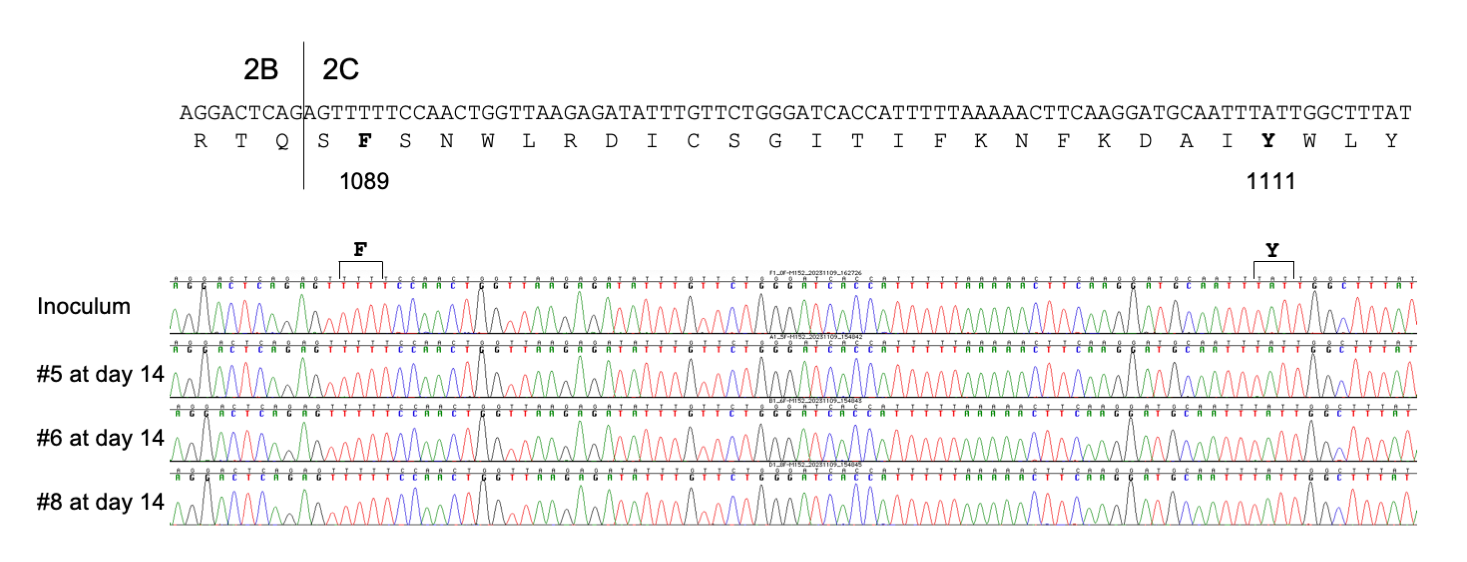

Supplement: S5 Fig — Viral RNA from the inoculated HAV, and from HAV recovered on Day 14 via fecal shedding (from three independent mice) following 14 days of once-daily administration of 15 mg/kg loxapine, was extracted, reverse transcribed, and amplified by PCR. The complete 2C-encoding region (and flanking sequences) of the amplified cDNA was sequenced. The nucleotide sequences of the complete 2C-encoding region (and the partial 2B-encoding region), along with the predicted amino acid sequences, are indicated above the electropherograms. (TIF) [file ppat.1012091.s005.tif]
